# The effect of steroid administration on fetal diaphragm function

**DOI:** 10.1186/s12884-022-05074-3

**Published:** 2022-10-12

**Authors:** Fatma Ozdemir, Gokhan Acmaz, Yusuf Madendag, Ilknur Col Madendag, Iptisam Ipek Muderris

**Affiliations:** 1 Department of Obstetrics and Gynecology, Erciyes University Faculty of Medicine, Yenidogan District, Turhan Baytop Street No:1, 38280 Kayseri, Melikgazi Kayseri, Turkey; 2 Obstetrics and Gynecology Department, Kayseri City Hospital, Kayseri, Turkey

**Keywords:** Betamethasone, Fetal diaphragm, Diaphragm thickness, Diaphragmatic excursion, Costophrenic angle

## Abstract

**Background:**

Antenatal steroid administrations lead to not only accelerated lung maturation, improved blood gas measurements but also lung dynamics and lung compliance. This study aimed to investigate structural and functional changes in diaphragm after antenatal steroid administration.

**Methods:**

The 79 volunteers were divided into 2 groups according to presence of preterm delivery. Betamethasone (Celestone^R^) 12 mg intramuscularly was routinely administered to pregnancies complicated with preterm delivery between 28th -34th weeks of gestation. Same dose was repeated 24 h later. In all patients, diaphragm thickness, diaphragmatic excursion and costophrenic angle were measured in both the inspirium and expirium stage of respiration. This is an observational cross-sectional study.

**Results:**

Diaphragm thickness, diaphragmatic excursion and diaphragm thickening fraction parameters were improved but costophrenic angle was not different 7 days after steroid administration. Diaphragm thickness, diaphragmatic excursion and costophrenic angle changes during inspiration and expiration stage after 7 days of betamethasone treatment reflects the effect of steroid administration on diaphragm muscle. Comparisons of the differences that occur after steroid rescue protocol were done by subtracting the diaphragm thickness, diaphragmatic excursion and costophrenic angle parameters before the treatment from the diaphragm thickness, diaphragmatic excursion and costophrenic angle parameters 7 days after steroid treatment respectively.

**Conclusion:**

Diaphragm became more mobile in patients with preterm labor, 7 days after steroid administration. This situation reflects positive effect of steroid administration on diaphragm function.

## Background

Uterine contractions accompanying cervical dilatation > 2 cm and cervical effacement ≥ 80% prior to 37th completed weeks of gestation is accepted preterm delivery and it is related with morbidity and mortality in infants [1]. Because neonatal death, perinatal death, intraventricular haemorrhage (IVH) respiratory distress syndrome (RDS), transient tachypnea (TT) of the newborn, requirement of neonatal intensive care unit (NICU) for observation or intervention and other co-morbidities are more common in pregnancies before 34th completed weeks of gestation, National Institutes of Health recommends antenatal steroid administration in 1994 [2].

Respiratory distress syndrome is important for neonatal health which is related with surfactant deficiency and inadequate anatomical lung formation [3]. Therefore antenatal steroid administration in patients with preterm delivery is related with increased surfactant production in fetuses. Moreover prenatal steroid administrations lead to not only accelerated lung maturation, improved blood gas measurements but also lung dynamics and lung compliance [4].

As the main muscle of human respiration, the diaphragm plays an important role in the pathophysiological process of respiratory failure. The diaphragm is the primary muscle of inspiration used in spontaneous breathing, so assessment of diaphragm dysfunction is pivotal in patients with respiratory failure [5].

In recent years, with the application of advanced ultrasound technology, increasing attention has been given to ultrasound evaluation of diaphragm function, including diaphragm thickening fraction (DTF), diaphragm displacement (DE) and lung ultrasound score (LUS). These indicators can be used to evaluate the alveolar ventilation insufficiency caused by alveolar collapse in critically ill patients during weaning, and they have certain predictive value for the weaning results of patients on mechanical ventilation[5].

In intrauterine life, fetal breathing movements are clearly not for purposes of gas exchange as that occurs via the placenta. In fact, fetal breathing movements consume oxygen that could otherwise be directed for tissue growth. Rather, fetal breathing movements serve the critical purpose of promoting the proliferation and differentiation of lung tissue [6].

Some neurochemical factors modulate fetal breathing movements in intrauterine life. For example, Prostaglandins (PG), specifically via PGE2, participate in the modulation of electroencephalogram (EEG) state and the suppression of fetal respiratory movements in utero [7].

Today we know that, corticosteroids inhibits prostoglandin formation and they cause adrenergically mediated vasoconstriction and non-competitive antagonism of vasodilation due to prostaglanin E and bradykinin [8].

Additionaly, as we known from literature, corticosteroids have a doping effect on sport performance. According the study by Vedhi et al., the use of corticosteroids has been known to result in increased mass gain with bovines [9].

So, it can be speculated that corticosteroids may act to increase fetal respiratory movements through prostaglandin inhibition and diaphragm muscle mass gain. And this can be objectively demonstrated by ultrasonographic examination of the fetal diaphragm. This study aimed to investigate the effect of steroid administration on diaphragm muscle function in patients with threatened preterm delivery.

## Methods

As a first step, approval for this study was obtained from the academic council of department. Then Ethical Committee of Erciyes University School of Medicine (No:2018/321) approved this study. Verbal and written informed consent form was signed by all volunteers. After necessary steps, this prospective controlled study was performed at the Department of Obstetrics and Gynecology clinic of Erciyes University, a tertiary referral center, between January 2019 and April 2020.

### Patients

We have two groups; first group consisted of 39 pregnant who are complicated with preterm delivery and second group consisted of gestational age matched 40 healthy pregnant without any complication. All of the 79 volunteers were monitored in our clinic from the beginning of pregnancy to birth. Patients who are referred from other clinics were not included this study.

All participants screened for fetal anomaly in the first and second trimester during pregnancy. Fetal gender determination was achieved during 21th -22th weeks of gestation by an obstetrician (F. O.). In case of suspicious results for aneuploidy in the first and second trimester screening tests or presence of fetal structural anomaly at the 21th -22th weeks of gestation via sonographic examination, we excluded volunteers from the study. We included only male fetuses to avoid changes in the neonatal outcome according to the gender. Thus, homogenization of the groups was achieved. All participants received scheduled cesarean section (CS) under spinal anesthesia due to their previous CS. So homogenization of the groups was achieved in terms of delivery and anesthesia type. During operation fetal arterial cord blood were obtained to investigate infants’ blood pH level, partial pressure of O2 and CO2, saturation of O2 and CO2 base excess and lactate levels. A pediatrician was examined newborns for NICU admission requirement, respiratory distress syndrome, APGAR scores, hypotonia, and hypoxia.

#### Definition of preterm delivery and steroid administration

The study group consisted of 39 patients between 28-34th weeks of gestation diagnosed as having preterm delivery during their first admission to our clinic. Patients with cervical dilatation > 2 cm and cervical effacement ≥ 80% or cervical length < 20 mm (mm) or cervical length between 20 and 30 mm with positive fetal fibronectin test before 34th weeks of gestation accepted as having preterm delivery [10]. And these patients formed the study group.

Among eligible participants who were diagnosed as preterm delivery between 28th -34th weeks of gestation received betamethasone (Celestone) 12 mg intramuscularly. Second dose of betamethasone (12 mg) was administered 24 h after first dose. 40 healthy, gestational age matched, pregnant woman without any complaining and administered our policlinic for routine control constituted control group. For matching the pregnant women in the study and control groups according to their gestational ages, each patient included in the study group was matched with a healthy, uncomplicated pregnant woman who did not receive any steroid or tocolysis in the same week as the week she was included in the study. The patients who formed the control group, were not given any steroids or any tocolytics.

All examinations were performed by an obstetrician (FO) blinded to the diagnosis of the patients reviewing recorded examination videos frame by frame.

### Exclusion criteria

**Fig. 1:**
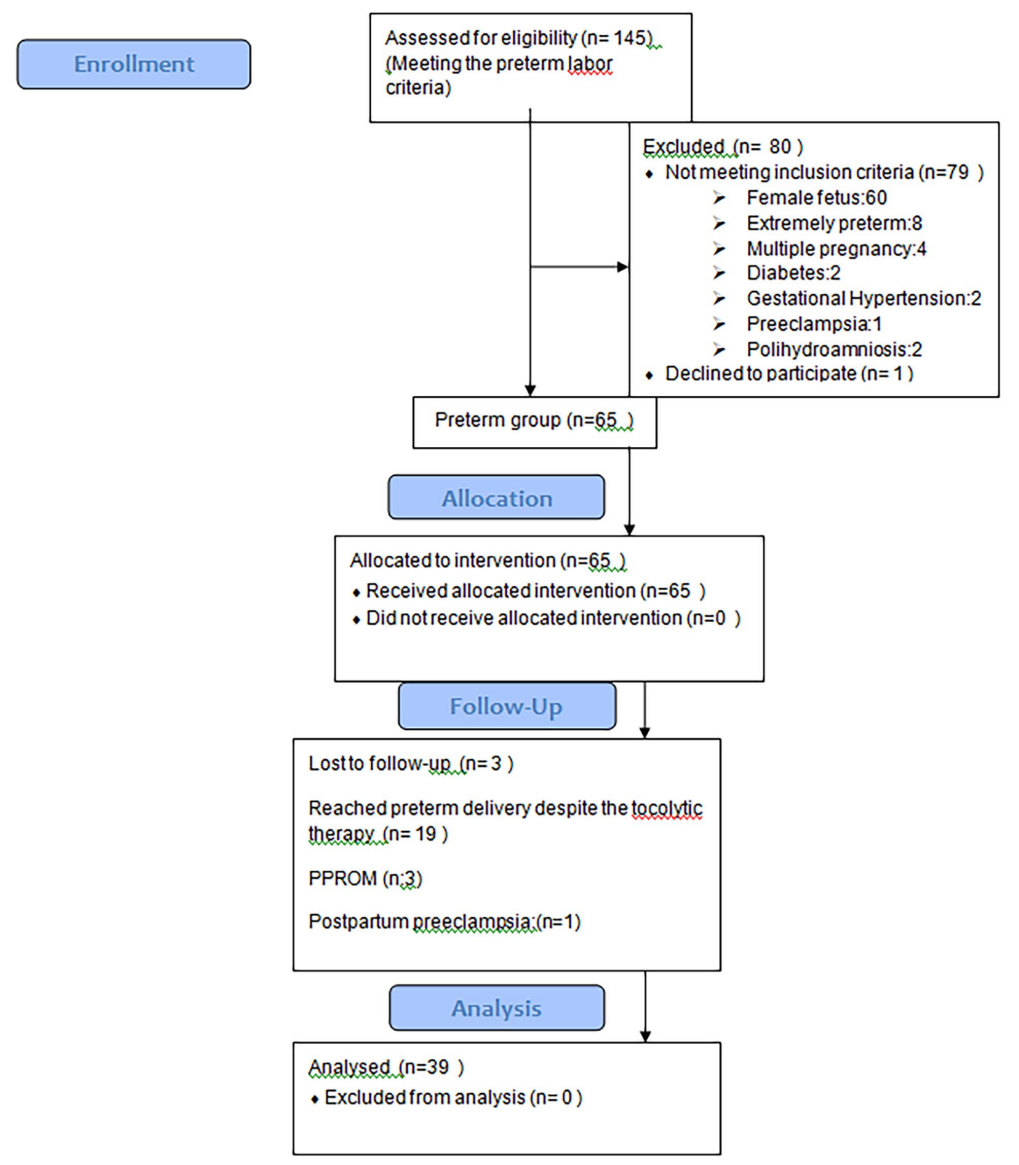
Flowchart of the study

Patients complicated with extremely preterm delivery (22–28 th gestational ages), membrane rupture, presence of CMV, toxoplasma, rubella, and syphilis antibody IG M, maternal fever or infections, twin or multiple pregnancies, presence of female fetus, suspicious first and second trimester screening test or fetal structural abnormalities, drug use (sedative, alcohol, tobacco, narcotic), presentation abnormalities (transverse oblique breech), urgent cesareans (fetal distress), patients complicated with gestational or pre- gestational diabetes, eclampsia, preeclampsia, gestational hypertension, amnion fluid abnormalities (oligohydramnios polyhydramnios) and chronic maternal diseases (liver, thyroid, kidney, and heart disease), were excluded from the study. Figure 1 represents enrollment, allocation and analyses of volunteers in the study.

### Evaluation of diaphragm muscle and time point of sonographic examination

In studies conducted in adult intensive care patients, two main ultrasonographic data were used as predictors of diaphragm function. The first is the “diaphragmatic excursion”, which measures the distance the diaphragm can move during the respiratory cycle, and the second is the “diaphragmatic thickening fraction”, which also reflects the change in diaphragm thickness during the respiratory cycle. Therefore, these two parameters were found suitable for evaluation in terms of demonstrating fetal diaphragm function. The costodiaphragmatic angle, on the other hand, was taken into consideration as an indirect finding of diaphragmatic mobility, as it would increase or decrease as the diaphragm moved [11–13].

Diaphragm images were recorded at two time-points. The first record was obtained at the first admission of patients and prior to steroid administration with preterm delivery. The second record was obtained 7 days after steroid administration. Data collected from neonates born in the early preterm period suggest that the benefit of antenatal corticosteroids is greatest among those who deliver 2 to 7 days after the first dose. Based on this information, ultrasonographic examination was planned to be performed on the 7th day after the first dose of steroid in order to capture and determine the maximum efficacy of the steroid [14].

Respiration consists of two phases; inspiration and expiration stages. Thus we recorded these stages and measurements were done on these records. In course of examination, obstetrician aimed to record horizontal section of the diaphragm. To achieve this goal she moved her probe on the maternal abdomen for detection of the fetus perpendicular to the left and right chest wall and below the costal margin, to observe the zone of apposition of the muscle below the costophrenic sinus. This plane allows viewing diaphragm in three layers; parietal pleura and peritoneum compose two echogenic parts and central part of diaphragm can be visualized as a non echogenic part. In course of peak inspiration; right diaphragm remains at higher position than left diaphragm due to the position of the liver. Thus both left and right diaphragms examined one by one and mean values of left and right diaphragm were expressed. None of the fetuses examined in the presence of gasping or ‘picket-fence’ breathing phase.

### Evaluation of Diaphragm thickening fraction, diaphragmatic excursion and costophrenic angle Parameters

During respiration diaphragm moves up and down in expiration and inspiration stages. Thus we recorded at least two respiration cycles on record system of Voluson E6 then measurements were done on these records. All measurements were performed in duplicate at different times by the FO to assess the reproducibility and intra-observer reliability of the findings. The average distance between maximal elevation point of diaphragm and the lowest point of diaphragm is defined as diaphragmatic excursion (DE). DE can be accepted as marker of the ability of diaphragm movement during expiration and expiration states of breathing. Moreover a formula was used to determine Diaphragm thickening fraction (DTF) (end-inspiration thickness – end-expiration thickness / end-expiration thickness ×100). DTF today accepted as diaphragmatic function marker and later costophrenic angle (CPA) was evaluated on video records both inspiration and expiration stages [15].

**Fig. 2:**
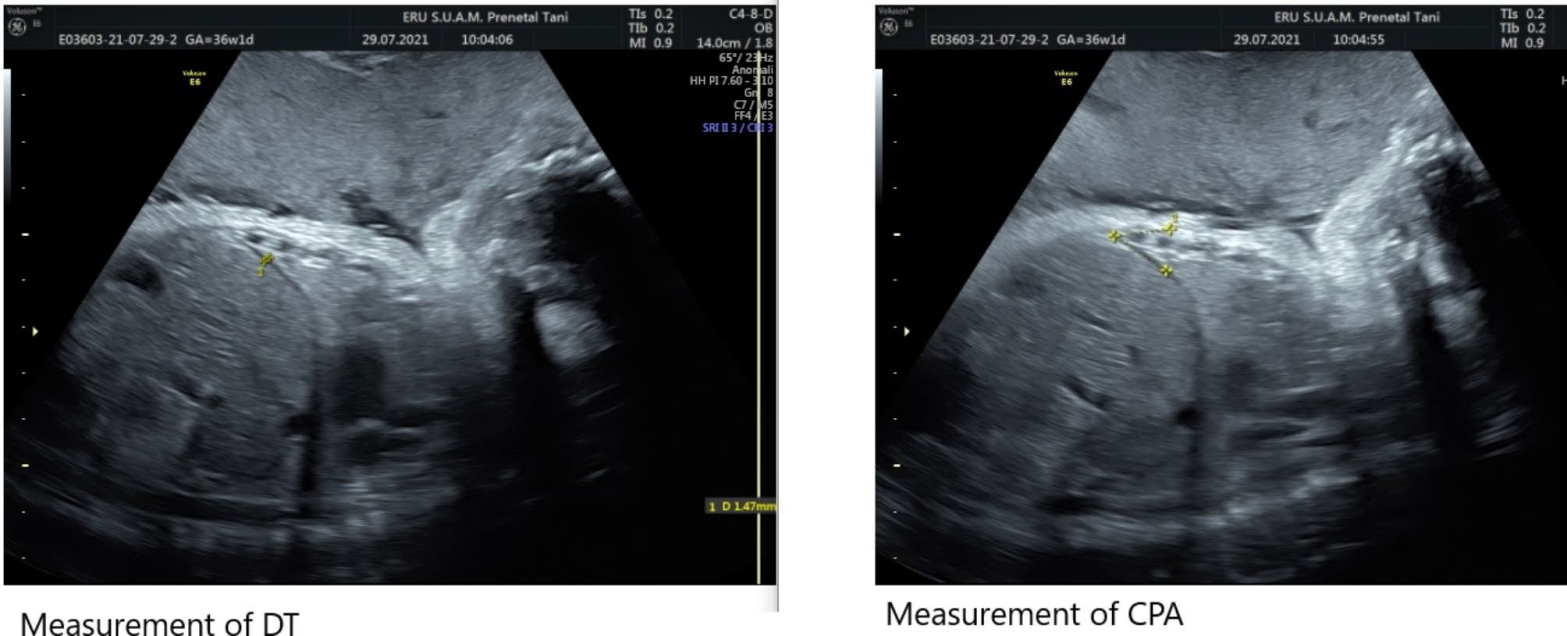
Ultrasonographic measurement of diaphragm thickness and costophrenic angle

Another important diaphragmatic function marker is Diaphragm thickening fraction (DTF) which was calculated using a formula (end-inspiration thickness – end-expiration thickness / end-expiration thickness ×100). Figure 2 represents measurement of diaphragm thickness and costophrenic angle by using ultrasound.

### Power analyses and statistics

#### Sample size

In pilot study, to determine the number of necessary volunteers we measured expiratory diaphragmatic thickness in preterm group before steroid administration (n: 15) and seven days after steroid administration. The mean value of expiratory diaphragmatic thickness was 1.46 ± 0.18 before steroid administration and 1.59 ± 0.25 after seven days steroid administration in preterm group. When we assumed alpha = 0.05 and power (1-β) = 0.8, 0.59 was calculated as effect size and 36 volunteers were required for both study and control group. However in the presence of 10% drop rate 40 volunteers were needed for each group.

#### Statistics

For the detection of data normality, Shapiro Wilk was used and Levene test was used to determine variance homogeneity. Mann – Whitney U test used for non parametric comparisons and t-test and z test were used for parametric comparisons. These comparisons were done via using PASW Statistics 18 program. When p value is under 0.05, probability is considered as statistically significant. Values are expressed as mean ± standard deviation, median (25th percentile – 75 percentile) or n (%).

For evaluating the reproducibility and intraobserver reliability of the findings; intraclass correlation method was used since the variables were continuous while performing the reliability analysis. Situations with an intraclass correlation value of 0,70 and greater were considered reliable. In other words, intraclass correlations with values of 0,70 and higher indicate the reliability of the measurements of the same observers, while values less than 0,70 indicate that the measurement are not reliable. In all cases, a two-way mixed-effects model was used, and since the observers were the same, the inter-observer errors were considered to be systematic. PASW Statistics 18 programme was used for all comparisons. p < 0.05 probability value was considered as statistically significant.

## Results

Ethnically all subjects were Caucasian and were followed until delivery. Demographic characteristics of study and control group recorded and we obtained umbilical cord arterial blood to investigate infants’ pH status, partial oxygen and carbon dioxide pressure, oxygen saturation and lactate levels during labor. These parameters of study and control group were illustrated in Table 1.

**Table 1:** Comparisons of patients between study and control group for demographic characteristics

	Study group (preterm delivery n = 39)	Control group (n = 40)	P - Value
Age (years)	29,41 ± 5,60	29,88 ± 5,62	0,714
Umbilical pH	7,37 (7,32 − 7,40)	7,36 (7,35 − 7,40)	0,553
Base excess	-1,50 (-2,70- -0,20)	0,58 (-0,80 − 0,90)	< 0,001
PO2 (mmHg)	92,30 (89,80–95,20)	90 (89–92)	0,002
PCO2 (mmHg)	41,60 (35–45,20)	47 (45–50)	< 0,001
SO2 (%)	95 (92,60–96,30)	96 (90,75–97)	0,712
Lactate (mmol/lt)	1,52 (1–2,36)	0,85 (0,60 − 1,18)	< 0,001
Labor time (days)	248 (238–260)	273 (266,25–280,75)	< 0,001
APGAR score first minute	8 (6–8)	8 (8–8)	0,001
APGAR score fifth minute	10 (8–10)	10 (10–10)	0,001
Neonatal Intubation	2 (5%)	0 (0%)	0,035
O2 treatment	10 (26%)	0 (0%)	0,002
NICU admission	12 (30,77%)	0 (0%)	< 0,001
Birth weight (gr)	2750 (2250–3150)	3225 (3100–3450)	< 0,001

As explained in methods section both preterm labor group and control group were homogeneous for age, gestational weeks and fetal biometry during examination. Although preterm group received tocolytic treatment, certain number of them did not reach term. Thus we detected significantly different base excess, PO2, PCO2, lactate, APGAR scores at the first and fifth minute, neonatal intubation, O2 treatment, NICU admission and birth weight between groups after delivery.

Among the 39 newborns in preterm delivery group, 2 of the neonatal received intubation, 2 babies received continuous positive airway pressure (CPAP), and 8 babies solely received oxygen treatment.

Both study and control groups were similar for gestational age at the first admission therefore all measurements were done at the first admission to hospital and 7 days after steroid administration. We illustrated these parameters in Table 2.

**Table 2:** Diaphragm thickness, Diaphragmatic excursion, costophrenic angle before and after steroid treatment both inspiration and expiration stages

	Study group (preterm delivery n = 39)	Control group (n = 40)	P - Value
DT during inspiration (Before steroid administration C 0) (mm)	1,72 ± 0,30	1,79 ± 0,31	0,345
DT during inspiration (7 days after steroid administration C 7) (mm)	1,78 ± 0,31	1,82 ± 0,31	0,570
DT during expiration (Before steroid administration C 0) (mm)	1,53 ± 0,27	1,55 ± 0,25	0,723
DT during expiration (7 days after steroid administration C 7) (mm)	1,60 (1,38 − 1,80)	1,58 (1,34 − 1,87)	0,676
DE (Before steroid administration C 0)	4,63 ± 0,86	4,73 ± 0,56	0,531
DE (7 days after steroid administration C 7)	4,87 ± 0,87	4,92 ± 0,60	0,769
CPA during expiration (Before steroid administration C 0) (degrees)	42 (35–50)	40 (36–44,25)	0,084
CPA during expiration (7 days after steroid administration C 7) (degrees)	43,50 (37–52)	41 (38–45)	0,080
CPA during inspiration (Before steroid administration C 0) (degrees)	56,82 ± 6,64	59,33 ± 6,47	0,094
CPA during inspiration (7 days after steroid administration C 7) (degrees)	58,96 ± 6,67	60,81 ± 6,51	0,216

DT, DE and CPA changes during inspiration and expiration stage after 7 days of treatment reflects the effect of steroid administration on diaphragm muscle. Comparisons of the differences that occur after steroid rescue protocol were done by subtracting the DT, DE and CPA parameters before the treatment from the DT, DE and CPA parameters 7 days after steroid treatment respectively. These changes were illustrated in Table 3.

**Table 3:** Changes of DT, DE and CPA parameters both inspiration and expiration stages between steroid day 7 and steroid day 0

	Study group (preterm delivery n = 39)	Control group (n = 40)	P - Value
DTF C0	11,39 (9,74 − 13,75)	15,26 (11,19–19,14)	0,009
DTF C7	11,11 (9,85 − 14,29)	13,86 (11,28 − 18,36)	0,015
DT change for inspiration between (Celestone day 7-Celestone day 0) mm	0,05 (0,04 − 0,08)	0.045 (0,02 − 0,05)	0,004
DT change for expiration between (Celestone day 7-Celestone day 0) (mm)	0,05 (0,03 − 0,07)	0,04 (0,01 − 0,05)	0,012
DE change (Celestone day 7-Celestone day 0) (mm)	0,25 (0,20 − 0,30)	0,20(0,10 − 0,30)	0,041
CPA change for inspiration between (Celestone day 7-Celestone day 0) (mm)	2 (1–3)	1,50 (1–2,75)	0,071
CPA change for expiration between (Celestone day 7-Celestone day 0) (mm)	2 (1–2)	2 (1–2)	0,689

Except for CPA we observed positive changes in DT, DE and DTF parameters 7 days after steroid administration

The intraobserver reliability of each parameter was given in Table 4.

**Table 4:** İntraobserver reliability of diaphragma measurements

	Intraclass correlation	%95 confidence interval	p-value
DT during inspiration (Before steroid administration C 0)	0,991	0,986-0,995	< 0,001
DT during inspiration (7 days after steroid administration C 7)	0,992	0,980-0,996	< 0,001
DT during expiration (Before steroid administration C 0)	0,997	0,988-0,999	< 0,001
DT during expiration (7 days after steroid administration C 7)	0,995	0,988-0,998	< 0,001
DE (Before steroid administration C 0)	0,993	0,990-0,996	< 0,001
DE (7 days after steroid administration C 7)	0,940	0,907-0,962	< 0,001
CPA during expiration (Before steroid administration C 0)	0,992	0,988-0,995	< 0,001
CPA during expiration (7 days after steroid administration C 7)	0,990	0,970-0,995	< 0,001
CPA during inspiration (Before steroid administration C 0)	0,993	0,989-0,996	< 0,001
CPA during inspiration (7 days after steroid administration C 7)	0,991	0,976-0,995	< 0,001

According to the Table 4; the parameters were found to be reliable and reproducible.

## Discussion

Nearly 10% of all births complicated with preterm delivery and 1 million newborns die due to preterm labor related complications like IVH, RDS, TT of the newborn, NICU admission, necrotising enterocolitis (NEC), patent ductus arteriosus and sepsis [16].

Today betamethasone is routinely used in the presence of preterm labor under 34th weeks of gestation for reducing neonatal morbidity and mortality [2].

In animal models the effect of steroids on lung maturation repeatedly studied and authors concluded that steroid administration to patients with preterm labor resulted increased surfactant production and corticosteroid exposure triggered a synchronous maturation response for most of the fetal organs and systems. Additionally it was speculated that glucocorticoids might have not only biochemical effects but also physiological and structural benefits [16].

Pew BK et al. pointed out that steroid administration in the presence of preterm delivery may lead to increased lung volume, lung porosity and airspace. These results have been shown in an ERK 3 null mouse model of respiratory distress syndrome after carrying out steroid injections via using micro computerized tomography [17].

One of the important points of our study is high resolution USG examinations of the fetal diaphragm enables detailed functional characterization of respiratory muscle. DT and DTF measurements may be accepted as marker of the diaphragm contraction efficiency [18].

Second, this study examined structural changes in diaphragm muscle after betamethasone rescue protocol which previous studies focused on histological changes. Additionally, the third strength can be accepted that this study carried out in human being during intrauterine life.

Patients who is planned liberation from mechanic ventilation, takes their own spontaneous breath if they have no diaphragm weakness or dysfunction. First breath of newborns mimics this situation and diaphragm ultrasound (DUS) can be used to define information about its morphology and function [19, 20].

Finding of study revealed that diaphragm became more mobile in patients with preterm labor, 7 days after steroid administration. This situation reflects positive effect of steroid administration on diaphragm function.

In a study of Lewis M I et al. authors investigated the effect of daily steroid use (for 21 days) on diaphragm muscle and diaphragm fatigue on hamster model. They pointed out that especially type II fiber atrophy and musculoskeletal fatigue occurs after steroid administration [21]. In another study Quatrocelli M et al. reported that dosing modulation resulted different steroid responses on sarcolemmal cells. They investigated a single pulse dose of steroid (intermittent) versus daily use of steroids in murine model of muscle injury. Daily steroid use promoted muscle atrophy plus repair of muscle on the other hand intermittent steroid use exert same effect without atrophy [22]. In an interesting study Pinet C et al. examined 12 cystic fibrosis (CF) patients who underwent lung transplantation and received steroid plus immunosuppressive regimen to inhibit rejection then compared them 12 healthy volunteers. This study focused on pulmonary function, diaphragm mass, diaphragm strength, abdominal muscle mass and abdominal muscle strength. According to their report thickness of external oblique, rectus muscles tended to be decreased in steroid treatment group additionally internal oblique and transverses abdominis muscles were not significantly different between groups. However diaphragm mass was 47% greater in steroid group than control group and concluded that the bulk of respiratory muscles in steroid received group was greater than control group on the contrary of this finding the bulk of abdominal muscles was greater in control group than steroid group. Their finding showed in human and transplantation patients but not in animals [23].

Similar to the study of Pinet C et al. we detected increased diaphragm thickness 7 days after steroid administration. This may be related to the use of betamethasone intermittently or betamethasone may increase diaphragm mass.

The diaphragm comprised of different structures. While central part of diaphragm consists of muscular tissue dominantly, peripheral part of diaphragm muscle consists of tendinous tissue [24].

Therefore, central part is more mobile than peripheral part of diaphragm. As a consequence of anatomic differences found in diaphragm muscle, CPA angle was similar between study and control group after steroid administration.

Conclusions: We can speculate that steroid administration has positive effect on function of diaphragm and morphology. Therefore, muscle strengthening agents may be used for preterm fetuses at the future. Evaluation of only vertex presentation, male fetuses complicated with preterm delivery can be accepted as study limitations. Thus there is a need for larger scale organizations.

## Data Availability

The data used to support the findings of this study are available on request from the corresponding author.

## Abbreviations

IVH: Intraventricular haemorrhage.
RDS: Respiratory distress syndrome.
TT: Transient tachypnea.
NICU: Neonatal intensive care unit.
CS: Cesarean section.
DTF: Diaphragm thickening fraction.
CPA: Costophrenic angle.
DE: Diaphragmatic excursion.
DT: Diaphragm thickness.
CPAP: Continuous positive airway pressure.
DUS: Diaphragm ultrasound.
NEC: Necrotising enterocolitis.
